# Setting multidisciplinary intervention goals for spinal muscular atrophy patients utilizing the international classification of functioning, disability, and health: a pilot study in a small sample sizes

**DOI:** 10.1007/s13760-025-02771-6

**Published:** 2025-03-26

**Authors:** Gabriele Giannotta, Marta Ruggiero, Greta Pirani, Maria Carmela Oliva, Camilla Ferrante, Antonio Trabacca

**Affiliations:** 1 Associazione “La Nostra Famiglia”– IRCCS “E. Medea” - Scientific Hospital for Neurorehabilitation - Unit for Severe Disabilities in Developmental Age and Young Adults (Developmental Neurology and Neurorehabilitation), Brindisi, Italy; 2 Scientific Institute I.R.C.C.S. “E. Medea”, Scientific Direction, Bosisio Parini (LC), Via Don L. Monza 20, 23842 Bosisio Parini (LC), Italy

**Keywords:** Spinal muscular atrophy, Patient-Centered care, International classification of functioning, Disability and health, Disability, Multidisciplinarity

## Abstract

**Background:**

Spinal Muscular Atrophy (SMA) is a genetic neuromuscular disorder causing progressive motor function loss, respiratory and swallowing impairments, and significant limitations in activities of daily living (ADLs) and social participation. While gene therapies have enhanced life expectancy, functional management remains crucial. Despite the International Classification of Functioning Disability and Health for Children and Youth (ICF-CY) being a valuable framework for patient-centered care, its integration in setting multidisciplinary rehabilitation goals for SMA patients is underexplored.

**Objective:**

This study aimed to assess the use of the ICF-CY framework in developing multidisciplinary intervention goals for SMA patients.

**Methods:**

A cross-sectional study involving 6 SMA patients was conducted at a pediatric neurorehabilitation hospital. Data were collected using a standardized ICF-CY-based case record form and analysed to identify patterns of impairment, activity limitations, and environmental factors.

**Results:**

Results demonstrated the multifaceted nature of SMA, with significant impairments in motor function, respiratory function, and swallowing, as well as limitations in activities of daily living and social participation. Environmental factors, particularly social support and accessibility, emerged as crucial determinants of overall well-being.

**Conclusions:**

The study underscores the importance of a multidisciplinary approach and the use of ICF-CY framework for developing comprehensive and patient-centered care plans for SMA patients.

## Introduction

Spinal muscular atrophy (SMA) is a genetic neuromuscular disorder characterized by the gradual deterioration of alpha-motor neurons of the anterior horn of the spinal cord [1, 2]. This progressive loss of motor neurons translates into a slow but relentless weakening and atrophy of skeletal muscles throughout the body [3]. While progress in SMA management has been remarkable due to gene-based therapies (GBTs), including RNA- and DNA-based treatments, which have transformed SMA into a treatable and non-life-threatening condition with significant clinical improvement, addressing the patients’ functional limitations remains a complex and essential aspect of care [4–6]. Disabilities of varying severity from case to case have become the central focus of care pathways in the era of GBT and significantly impact patients’ daily lives [7]. Addressing disability remains an unmet need, with the additional challenge of evaluating residual disability through more precise and targeted measures. SMA presents a complex and heterogeneous clinical phenotype, primarily characterized by progressive skeletal muscle weakness and atrophy [8]. Specifically, deficits in motor function contribute to significant limitations in mobility-related activities, including ambulation, stair climbing, and transfers [2]. Furthermore, respiratory muscle weakness can compromise ventilation, potentially necessitating ventilator support [9]. Additionally, speech and swallowing difficulties can arise due to weakness in the relevant musculature [10–12]. Chronic fatigue is another frequent consequence, limiting energy levels and impacting activities of daily living (ADLs) [13]. Finally, joint contractures can develop as a result of muscle weakness, further restricting range of motion and function [14].

The functional limitations associated with SMA, including impairments in mobility, self-care tasks, and communication, exert a cascading effect on various aspects of daily life [15]. This can significantly reduce a patient’s independence, necessitating assistance with ADLs [16, 17] and impeding social interaction and activity participation, which can potentially lead to frustration, isolation, and depression [18]. The potential contribution of cognitive impairments to the presence of these dynamics is still under investigation [19, 20]. Family, caregivers, and a supportive environment play a crucial role in mitigating these challenges and promoting overall quality of life for SMA patients [17, 21]. Optimizing health and well-being in this population requires a comprehensive therapeutic program, tailored to address their functional limitations across all aspects of life [6]. This multifaceted strategy integrates physical therapy to bolster muscle function and flexibility, occupational therapy to empower independence in daily activities, and respiratory and speech therapies to address breathing, swallowing and communication challenges [5]. Assistive devices further enhance mobility and independence [15]. Finally, recognizing the emotional difficulties associated with SMA, psychological support could play a pivotal role in managing the psychological impact of the disease [22].

In this context, establishing appropriate and measurable treatment goals is essential for optimizing patient outcomes [23]. Current assessment measures for this patient population predominantly concentrate on isolated aspects of their functioning, such as motor performance, psychological well-being, activities of daily living, or quality of life [24, 25]. While these measures yield clinically valuable insights, they do not necessarily align with goals that truly matter to patients [26]. To guide goal setting more effectively, it would be beneficial to target outcomes that have a tangible impact on patients’ day-to-day lives or overall quality of life [24]. The International Classification of Functioning, Disability and Health (ICF) framework provides a standardized approach to evaluating a patient’s health and functioning across various domains [27]. This comprehensive model considers body functions and structures, activities and participation, and environmental factors that influence health. Despite its established benefits, the integration of the ICF model into goal-setting practices for SMA patients’ remains limited [6]. This pilot study on an initial small group of patients with SMA aims to explore the advantages of using the ICF framework for Children and Youth (ICF-CY) to develop patient-centered and clinically relevant goals in the SMA population, as well as to identify and illustrate the multidisciplinary nature of data collected within the ICF framework.

## Methods

### Study design and population

This pilot, cross-sectional study was conducted at the Pediatric Neurorehabilitation Hospital in Brindisi, Italy (Scientific Hospital for Neurorehabilitation - Unit for Severe Disabilities in Developmental Age and Young Adults, Associazione “La Nostra Famiglia”– IRCCS “E. Medea”). The study aimed to investigate management interventions for six consecutively admitted patients with Spinal Muscular Atrophy (SMA), all of whom had confirmed genetic diagnoses and were receiving SMN-targeted disease-modifying therapies (DMTs). Given the rarity of SMA, with an estimated global incidence of approximately 1 in 10,000 live births [5], and feasibility constraints, this cohort included all eligible patients admitted to the center between January and June 2024. The pilot design allowed for an assessment of the practical application of the ICF-CY framework in clinical care within this small cohort, rather than drawing statistically generalizable conclusions. Inclusion criteria entailed patients with a confirmed diagnosis of SMA, receiving gene therapy, and aged between 6 and 17 years. Exclusion criteria included patients with other neuromuscular or genetic disorders, those not receiving gene therapy, and patients with cognitive impairments preventing effective participation in the study. The study protocol was approved by the local ethics committee and informed consent was obtained from all parents of participants. Additionally, assent was obtained from children before collecting data. All procedures performed in this study were in accordance with the ethical standards of the 1964 Helsinki Declaration and its later amendments.

### Measures

The case record form used in this study included a standardized set of questions based on the ICF. The ICF’s general model marks a significant shift in health description, focusing on individuals’ functions, skills, and abilities rather than just limitations [27].

It provides a valuable framework for understanding health, complementing diagnostic systems, and considering the individual’s physical, social, and psychological environment for a more comprehensive assessment of disability [28].

**Fig. 1:**
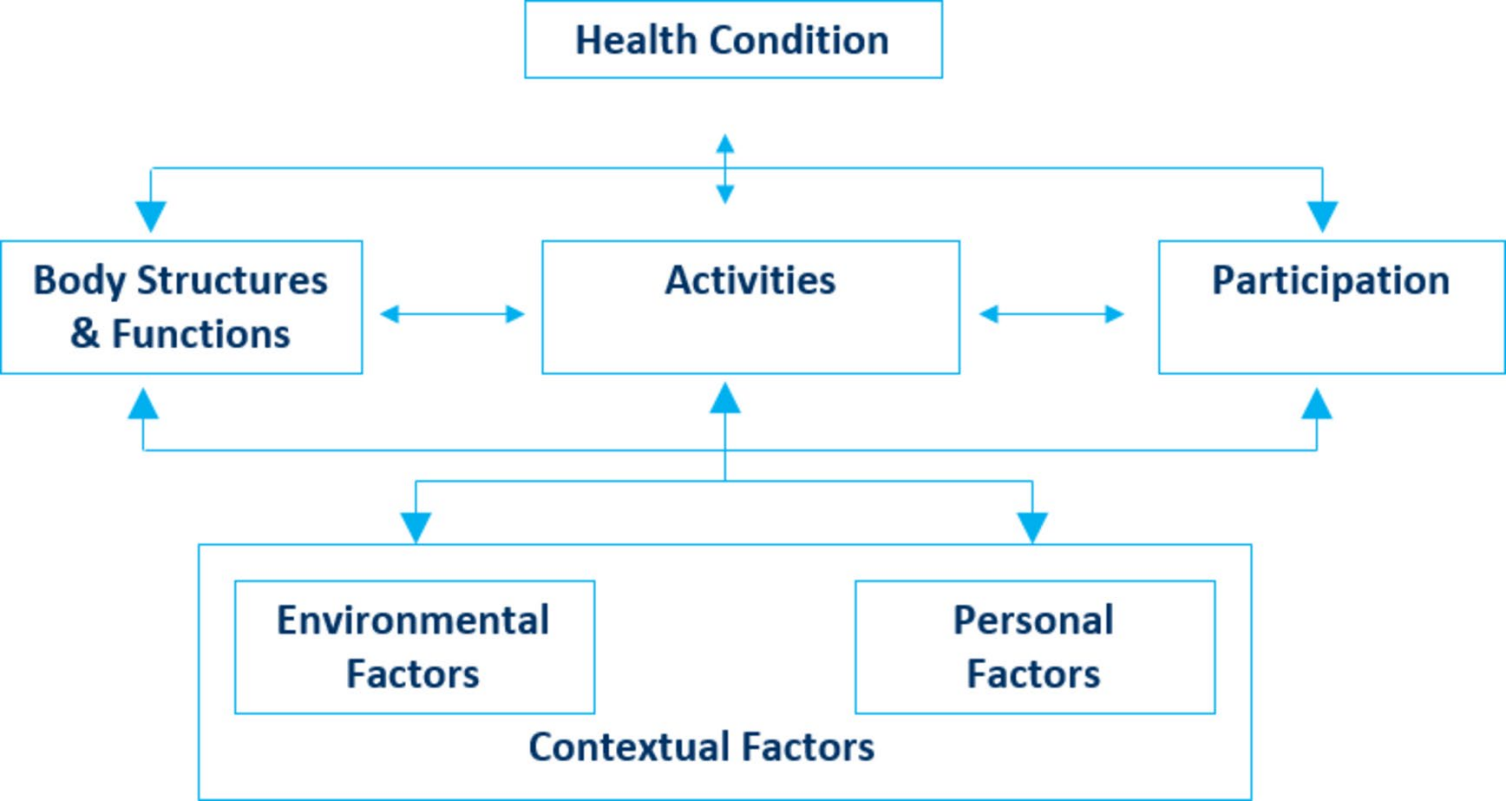
The conceptual framework of disability– International Classification of Functioning, Disability and Health (ICF) - World Health Organization (WHO)

This model consists of two parts, each encompassing two distinct aspects. Part 1 focuses on functioning and disability, covering the components “Body Functions” (b), “Body Structures” (s), and “Activities and Participation” (d). Part 2 addresses contextual factors and includes the components “Environmental Factors” (e) and “Personal Factors” *(*Fig. 1*)*. Each of them contributes to a nuanced understanding of health, transcending the identification of diagnoses provided by traditional systems [6].

The ICF-CY assessments used in this study were based on the validated version described by Leonardi et al. (2016) [29], based on the E. Bjorck-akesson version [30], ensuring alignment with internationally recognized coding standards for functional assessment.

This structured operational framework was designed to describe and measure health and functioning in children and youths from birth to 17 years and 11 months of age. The version of the form used in this study consisted of three parts, selected by the aforementioned team. Part 1 focused on functioning and disability, encompassing the components “Body Functions” (1a) and “Body Structures” (1b). Part 2 addressed “Activities and Participation”, and Part 3 assessed “Environmental Factors”.

Interviewers received training on recording limitations or impairments, differentiating those directly linked to the hospitalization and categorizing them as present or absent.

### Data collection and quality assurance

To ensure data quality, the study employed a standardized data collection protocol. A team of two healthcare professionals conducted interviews: a physiatrist and a neurologist. In order to guarantee the consistency and accuracy of data collection, the physiatrist and neurologist conducting the assessments underwent a structured one-day training session on the ICF-CY framework. This training covered theoretical principles, coding guidelines, and practical application exercises, supplemented by a standardized reference manual. Prior to each interview, the patient’s medical record was reviewed to gather relevant background information. If the patient was unable to provide certain details, healthcare professionals from the relevant ward or family members were consulted to supplement the data. Following each interview, the researcher examined the completed data collection form to ensure clarity and completeness. Any unclear statements were corrected, and additional comments were added for further clarification. Finally, all data forms underwent a secondary review by a separate individual to verify completeness and plausibility of the recorded information. This multi-step approach ensured data consistency and minimized the risk of errors or missing information.

### Post-Data collection expert review and treatment plan development

Following data collection, a team of experts from the study institution (neurologist, physiatrist, cardiologist, psychotherapist, physiotherapist, speech therapist, and occupational therapist) convened to review the ICF-CY profiles of each patient. Through collaborative discussions, the team identified areas requiring the pursuit of new or more tailored objectives. Based on these insights, each professional collaborated with the patient and their family to establish personalized treatment goals aligned with their specific needs. This multidisciplinary approach ensured comprehensive and patient-centered care.

**Fig. 2:**
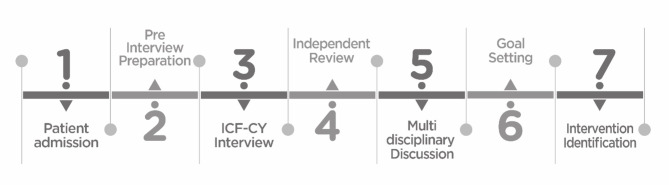
Process of patient assessment and intervention identification

The assessment phase and the development of the therapeutic plan are illustrated in Fig. 2.

### Data analysis

The expert team collected ICF-CY codes positively flagged as “problem presence”, regardless of their qualifier. Additionally, they determined that reporting ICF codes present in at least half of the patients would ensure the inclusion of only clinically impactful domains. Moreover, given the qualitative nature of the data collection in the “Environmental Factors” section, where only qualifiers are assigned, the authors have agreed to consider only scores of 3 and 4, indicating severe and complete presence of barriers, and + 0; +1, indicating no or slight presence of facilitation as indicators of the presence of the problem. Absolute and relative frequencies were used to quantify the International Classification of Functioning, Disability, and Health (ICF) fields representing areas of intervention needs. Absolute frequency refers to the number of participants within the sample reporting needs in a specific ICF-CY field. Relative frequency represents the proportion of participants reporting needs in that field compared to all categories. To analyze the association between perceived impairments and limitations in functioning, and the prevalence of intervention needs, we calculated the absolute and relative frequencies of participants reporting impairments or limitations within each ICF-CY category. Additionally, we determined intervention status (received intervention or not) for each category.

Furthermore, we analyzed the distribution of codes identified as problematic across the ICF-CY components while accounting for differences in the number of section codes. To achieve this, we examined the frequencies, defined as the number of patients reporting criticalities, for each section code within each component. For each component, we calculated the total frequency by summing the frequencies of all its section codes. This total was then normalized by dividing it by the number of section codes within the component, yielding a mean frequency per component. This normalization enabled a standardized comparison of criticalities across components.

In this case, to ensure uniform normalization relative to the total number of section codes in each component, frequencies of 0, 1, and 2 were included in the calculation.

## Results

Sample characteristics are shown in Table 1. ICF-CY codes and their corresponding descriptions, present in at least half of the patients, are reported in Table 2.

**Table 1:** Sample characteristics

Patient	Sex	SMA type	Age	Ambulant	Nutrition	Gene therapy
C.M.	F	2	17	No	PEG	Risdiplam
B.E.	F	2	17	No	Solid	Risdiplam
R.L.	F	2	16	No	Solid	Risdiplam
M.D.	F	3	17	Yes	Solid	Nusinersen
T.S.	F	2	17	No	Solid	Risdiplam
M.G.	M	3	15	Yes	Solid	Risdiplam
% - Mean	83.33% F 16.67% M	66,67% SMA2 33,33% SMA3	16.50 (S.D. 0.84)	66,67% No 33,33% Yes	83.33% Solid 16.67% PEG	83.33% Risdiplam 16.67% Nusinersen

**Table 2:** ICF-CY based equipe’s management of SMA patients

ICF code	Description	*n* impaired (% of impaired)	Goal setting	Intervnetion Identified
*ICF Domain: Body Functions*				
b134	Sleep functions	4(66.67%)	Improve sleeping setting (pre-bed routine, comfort, ecc)	PN
b280	Localized pain	3 (50%)	Pain Assessment	PED
b440	Respiration functions	5(83.33%)	Respiratory Assessment. Promote airway clearance	PUL, PED, RT
b510	Ingestion functions	4(66.67%)	Swallowing Assessment. Promote swallowing functions	DT
b525	Defecation functions	3 (50%)	To monitor the quality of excretory functions	PED
b710	Mobility of joint functions	6(100%)	Prevent ankyloses	PT
b730	Muscle power functions	6(100%)	Increase exercise tolerance	PT
b735	Muscle tone functions	6(100%)	Prevent contractures, weakness and immobility	PT
b750	Motor reflex functions	6(100%)	Control reflex functions	Phy, PN, PT
b760	Voluntary movement control and coordination functions	4(66.67%)	Improve voluntary movement control	PT
*ICF Domain: Body Structures*				
s430	Structure of respiratory system	4(66.67%)	Periodic respiratory system assessment	PUL, PED, RT
s710	Structure of the head and neck region	3 (50%)	Periodic head and neck assessment	PN, Phy, PT, OT
s730	Structure of upper extremity	4(66.67%)	Periodic upper extremity assessment	PN, Phy, PT, OT
s750	Structure of lower extremity	5(83.33%)	Periodic lower extremity assessment	PN, Phy, PT, OT
s760	Structure of trunk	5(83.33%)	Periodic structure of trunk assessment	PN, Phy, PT, OT
*ICF Domain: Activities and Participation*				
d170	Writing	5(83.33%)	Improve writing	OT
d230	Daily Life Activities	5(83.33%)	Expand the range of accomplishable common activities	OT, Psy
d330	Speaking	3 (50%)	Improve speaking	DT
d410	Changing basic body position	6(100%)	Support changes in position	PT, OT
d415	Maintainig a body position	4(66.67%)	Improve sitting, standing, head position	PT, Phy
d440	Fine hand use (picking up, grasping)	5(83.33%)	Improve fine hand functions	PT
d445	Hand and arm use	5(83.33%)	Improve hand and harm function	PT, OT
d450	Walking	5(83.33%)	Improve walking ability and dynamics	PT, Phy
d510	Washing oneself	5(83.33%)	Facilitate self-care	OT
d530	Toileting	4(66.67%)	Facilitate self-care	OT
d540	Dressing	5(83.33%)	Facilitate self-dressing	OT
d550	Eating	5(83.33%)	Improve eating ability	DT, OT
d6	Participation in household activities	4(66.67%)	Coach patient & family on maximizing shared housekeeping time	Psy
d9	Engagement in social life outside the family	4(66.67%)	Develop interpersonal skills	Psy
*ICF Domain: Einveronmental Factors*				
e115	Products and technologies for personal use in daily living	3 (50%)	Lightweight manual or electric wheelchair	OT
e125	Products and technologies for communication	3 (50%)	Eye tracker communicator	OT
e230	Natural events	4 (66.67%)	Properly equipped environment	OT
e325	Support of acquaintances, peers, neighbours and community members	3 (50%)	Community engagement	SW
e330	Support of people in positions of authority	4 (66.67%)	Community engagement	SW
e350	Support of pets	3 (50%)	Pet-therapy program	Specialized Psy
e430	Attitudes of people in positions of authority	3 (50%)	Community engagement	SW
e540	Transportation services, systems and policies	4 (66.67%)	Community engagement	SW

PN: pediatric neurologist; Phy: physiatrist; PUL: pulmonologist; PED: pediatrician; PT: physical therapist; RT: respiratory therapy; OT: occupational therapist; DT: dysphagia therapists; Psy: psychologist; SW: Social Worker

**Fig. 3:**
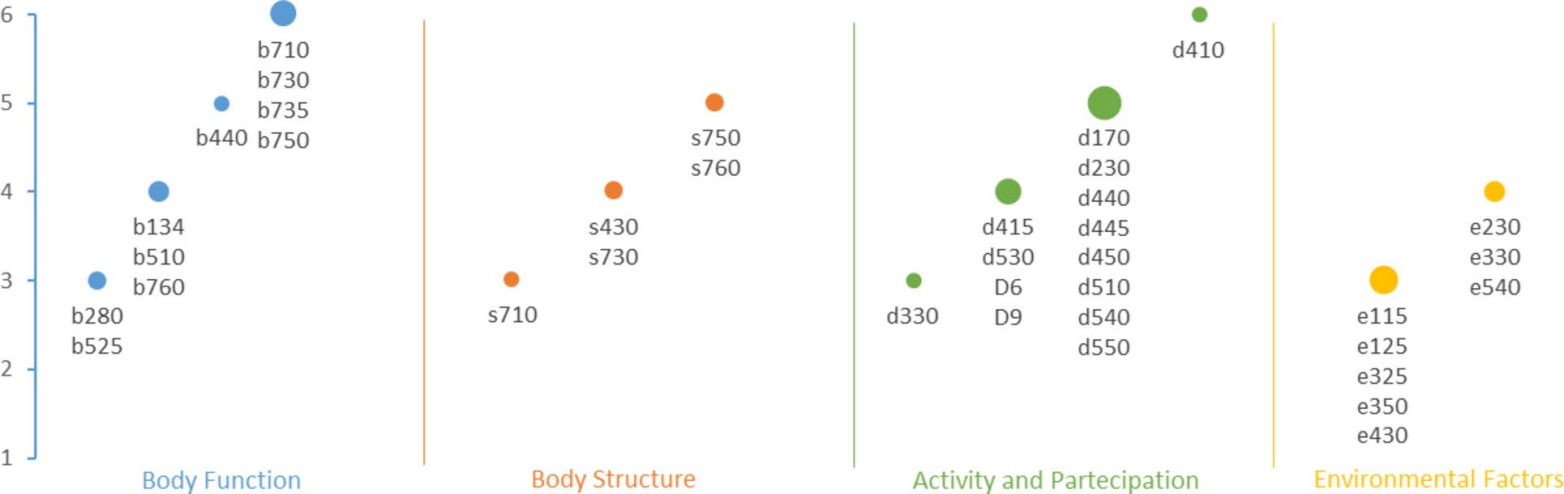
Absolute frequency of ICF - CY sections codes– The graph displays the recurrence of specific codes within our cohort of six patients, represented on the y-axis. The size of each point increases proportionally to the number of distinct codes present within each component

Absolute frequency of body function section codes is shown in Fig. 3. Motor function emerged as a dominant area of impairment, with all patients experiencing limitations in joint mobility, muscle strength, muscle tone, and motor reflexes. Furthermore, a significant proportion struggled with voluntary movement control and coordination. Respiratory function was another significant area of concern, with 83% of patients reporting issues with respiration, including abnormalities in rate, rhythm, and depth. Swallowing and digestion were also adversely affected; 67% of patients reported difficulties with ingesting solids or liquids, while 50% experienced challenges with defecation. Pain was a prevalent issue, with half of the patients experiencing localized or generalized pain. Beyond motor function, respiratory issues, and digestive difficulties, SMA patients face a broader spectrum of limitations. Some patients reported difficulties with sleep (including onset, maintenance, quality, and cycle), psychomotor function control, and basic cognitive functions. Additionally, vision issues, speech production problems, and involuntary movements were noted in a smaller proportion of patients.

In the body structure section (Fig. 3), the data revealed significant structural impairments, primarily affecting the musculoskeletal system. Half of the patients exhibited impairments in the bones, joints, and muscles of the lower extremities, while smaller percentages reported structural issues in the upper extremities and head and neck regions, respectively. These structural abnormalities resulted in considerable limitations in mobility, range of motion, and overall physical function. Respiratory structures were also frequently impaired, with 80% of patients presenting structural abnormalities in the respiratory system. Additionally, 20% of patients reported structural issues in the bones, joints, and muscles of the trunk, while 20% and 10% experienced structural abnormalities in the cardiovascular system and the structures of the eye or ear, respectively.

Figure 3 also illustrates the absolute frequency of activity and participation section codes. SMA significantly affects patients’ ability to perform daily activities and participate in social life. All patients reported limitations in performing basic activities of daily living (ADLs), such as washing, dressing, and eating. Furthermore, 80% of patients experienced difficulties with mobility, including changing body position, maintaining posture, walking, and using stairs. Social participation was markedly limited, with most patients reporting difficulty engaging in activities outside the family, such as school, community involvement, and leisure pursuits. Additionally, 50% or more of patients reported difficulties with managing bodily functions, experiencing impairments in fine motor skills, in activities like using hands, fingers, and thumbs. Communication impairments was reported to affect both comprehension and expression in one-third of our sample.

The analysis of environmental factors (Fig. 3) highlighted a range of challenges that significantly influenced treatment decisions and outcomes for patients with SMA. Among these, natural events (e.g., extreme weather) were identified as a barrier by 66.7% of participants. These events often disrupted patient transfers, limited attendance at therapy sessions, and generally decreased comfort. For instance, one family reported that heavy rainfall frequently made it impossible to navigate their wheelchair-equipped vehicle on certain roadways, forcing them to postpone scheduled hospital visits. Such postponements curtailed both consistency and momentum in the rehabilitation process.

**Fig. 4:**
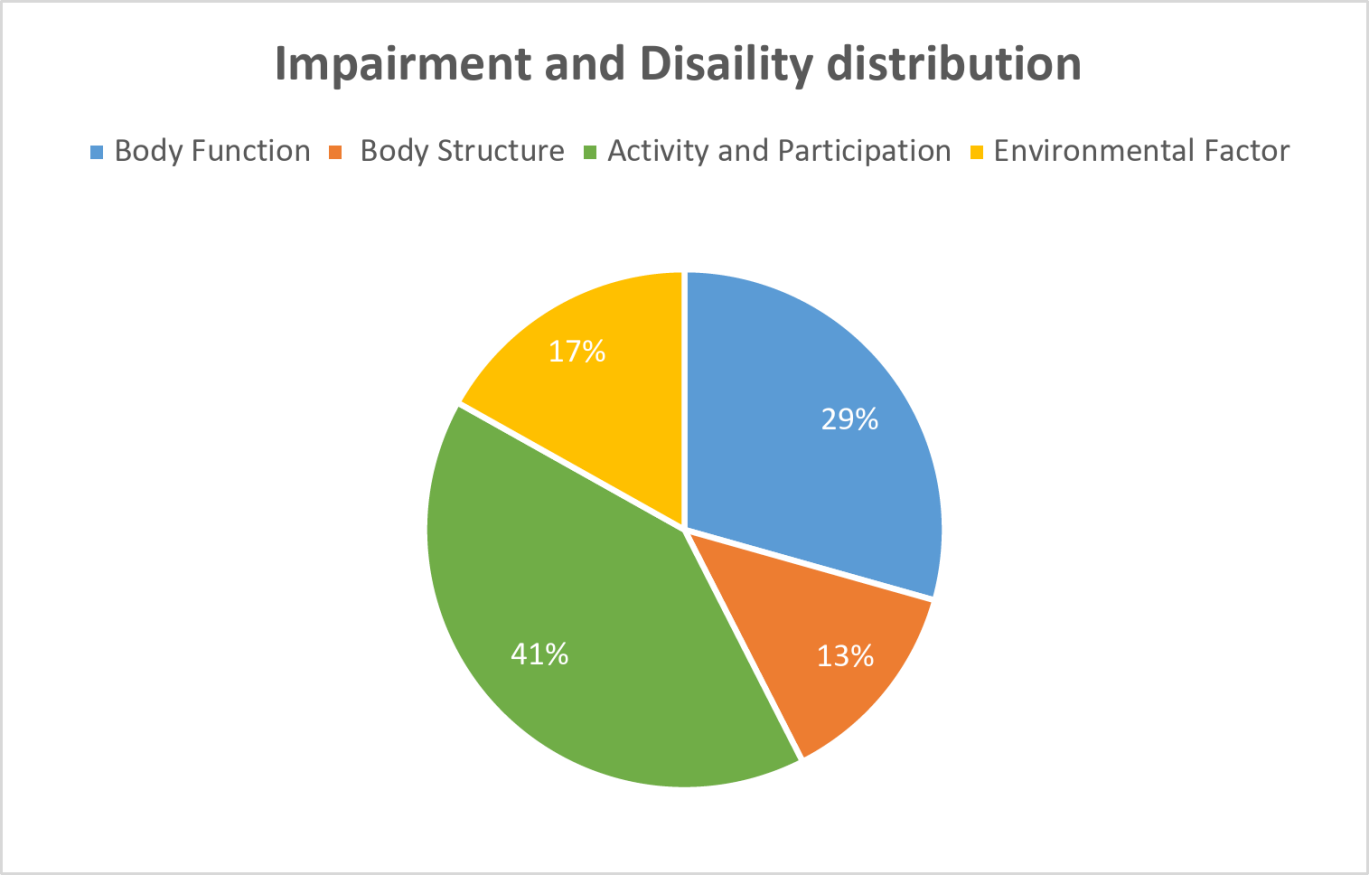
Relative frequencies of affirmative responses regarding impairment and disability codes across ICF-CY components

Lack of accessible transportation, also identified by 66.7% of participants, emerged as another critical barrier to mobility and participation in daily life. For families in rural areas, the absence of specialized public transport meant relying entirely on private vehicles or on community-based resources with limited availability. One parent of a “sitter” SMA patient, described how scheduling adapted transport for any non-emergency trip involved long wait times, which not only affected their child’s therapy schedule but also reduced opportunities for social and recreational activities. In one case, the ICF-CY identified severe limitations in mobility (d450), along with constraints in products and technology for personal use in daily living (e115). This prompted the multidisciplinary team to collaborate with the patient’s family on acquiring new assistive equipment and modifying existing devices, such as adjustable seating and supportive aids, to enhance functional independence in daily activities. Additionally, limited access to assistive technologies (50%) encompassing daily living and communication tools further hindered functional independence. Some families reported that the process of obtaining specialized seating or communication devices was prolonged due to funding constraints and administrative hurdles, which delayed the integration of these aids into the child’s daily routine. Figure 4 illustrates the relative frequencies of affirmative responses regarding impairment and disability codes across ICF-CY components.

Taken together, Activity and Participation codes remained the most frequently reported as problematic, but Environmental Factors played a pivotal role in either intensifying or alleviating these activity limitations. The concrete examples outlined above underscore how environmental constraints can impede the day-to-day implementation of clinical plans, thereby emphasizing the necessity of addressing these factors as part of comprehensive SMA care.

**Fig. 5:**
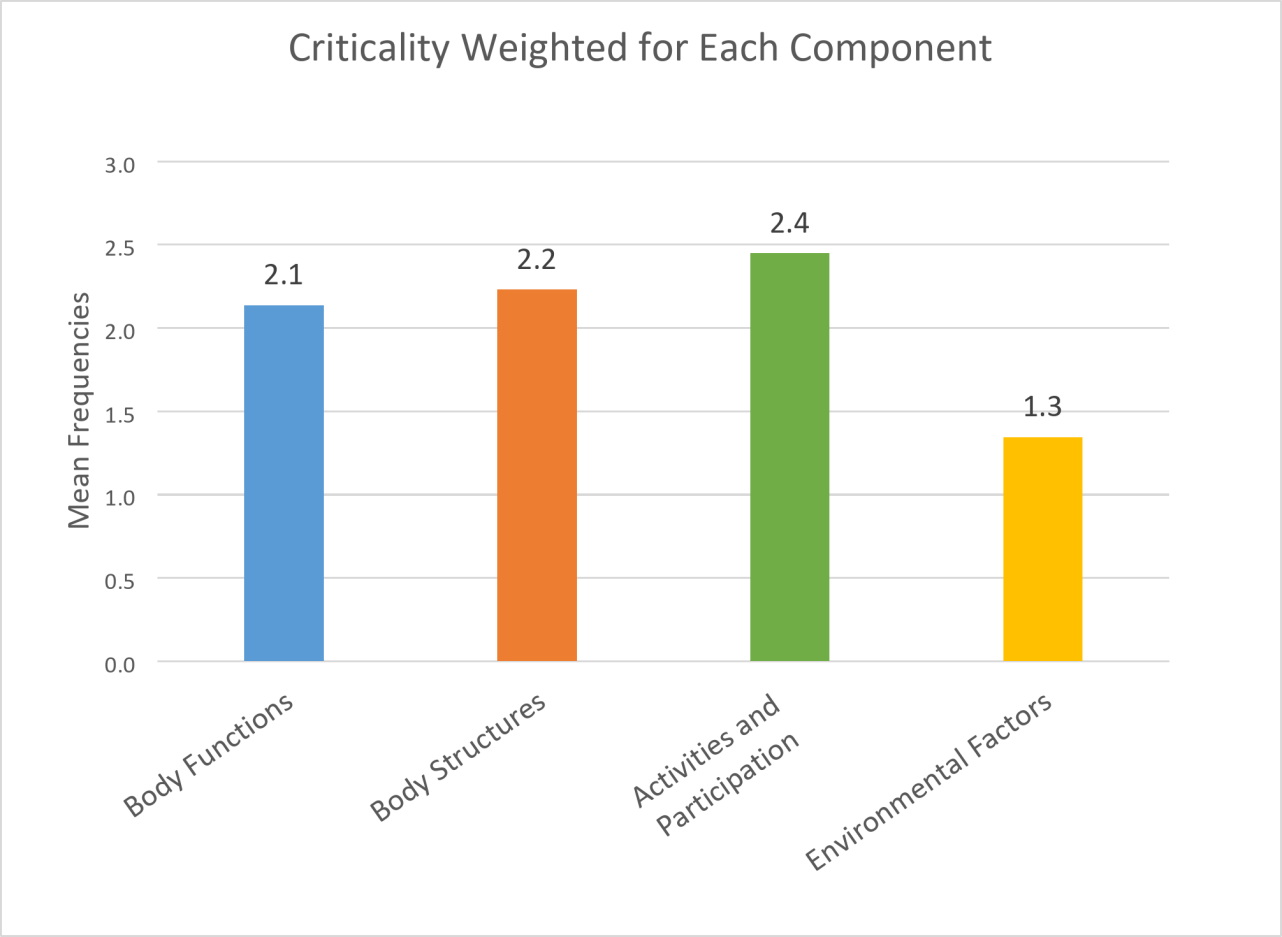
Bar chart of the Mean frequencies for each ICF-CY components

The bar chart *(*Fig. 5*)* depicts the mean frequency for each ICF-CY component, calculated by summing the frequencies of reported criticalities and normalizing them by the number of section codes within each component. A higher mean frequency indicates that, regardless of differences in the number of section codes, a greater proportion of patients experienced difficulties in that component. As illustrated in Fig. 5, Activities and Participation exhibited the highest mean frequency (2.4), followed by Body Structures (2.2) and Body Functions (2.1). In contrast, the Environmental Factors component showed the lowest mean frequency (1.3).

Based on the ICF-CY assessment findings, the multidisciplinary team formulated tailored therapeutic goals targeting each patient’s key challenges. For instance, when limited joint mobility emerged as a concern, the physiotherapist collaborated with the occupational therapist to introduce appropriate exercises and adaptive strategies for daily living. If communication or swallowing issues were identified, the speech therapist implemented specific interventions to address these needs. By drawing on each specialist’s expertise, the team ensured the goals were both practical and achievable, ultimately supporting improved function and well-being.

A comprehensive overview of all the data presented is shown in Table 2.

## Discussion

SMA presents a complex spectrum of challenges for patients, impacting not only their physical abilities but also their overall well-being. This analysis of patient data using the ICF-CY framework highlighted the multifaceted nature of these impairments and underscored the importance of a comprehensive assessment approach and setting broader therapeutic goals.

The ICF-CY has proven valuable in documenting the range of characteristics and consequences experienced by children with various pediatric conditions [31, 32]. It facilitates the creation of a functional profile for each child with special needs, further clarifying the interaction between child and environment that influences their functioning [28].

Given the extensive and complex impairments observed in SMA patients, the ICF-CY offers a critical tool for systematic evaluation across body functions and structures, activities and participation, and environmental factors. Although traditional assessments in SMA often center on specific outcomes such as motor function or quality of life, the ICF-CY adopts a more holistic lens. For example, various patient- or caregiver-reported outcome measures (PROMs) and functional scales (e.g., the Pediatric Quality of Life Inventory or the Barthel Index) may capture discrete domains like fatigue, independence in activities of daily living, or caregiver burden [33, 34]. However, these tools do not always provide a unified view of how contextual and psychosocial elements intersect with physical impairments. By contrast, the ICF-CY framework helps integrate all these dimensions, guiding more comprehensive goal-setting, treatment and rehabilitation planning [6].

The intricate nature of SMA necessitates an equipe-based model, where each healthcare professional contributes specialized expertise [7]. This could become analogous to disentangling a complex web of challenges. While motor function limitations are a hallmark of SMA, the condition also presents with respiratory dysfunction, swallowing difficulties, and potential psychological distress. A limited patient perspective hinders effective addressal of the full spectrum of experienced impairments. For instance, a paediatrician plays a crucial role in monitoring growth and development, while a respiratory therapist ensures optimal respiratory function. Physical therapists address limitations in mobility, psychologists provide support for emotional well-being, and dysphagia therapists manage swallowing complications [6].

Each specialist brings a distinct perspective, and the ICF-CY framework facilitates a unified understanding of the patient’s condition, enabling the equipe to develop a comprehensive care plan [27].

Our data analysis revealed that, within our sample, the Activities and Participation domain was the most affected, both in terms of relative frequency and mean frequency. This finding remains valid even after accounting for potential statistical bias introduced by the larger number of codes associated with this domain, reinforcing its significance in assessing functional limitations. The persistent prominence of Activities and Participation suggests that, beyond structural impairments, restrictions in daily activities and social engagement represent the most pressing challenges for our patients group. This underscores the necessity of targeted interventions that enhance functional autonomy and participation in meaningful activities, rather than focusing solely on structural impairments [35].

Interestingly, when considering the potential bias introduced by domain-specific code distribution, our findings indicate that Environmental Factors were less influential than Body Structures in our cohort. Although the Environmental Factors domain encompasses a broad set of contextual influences, it appears that structural impairments, despite being assessed through a more limited number of codes, played a more significant role in shaping patient difficulties. This suggests that even though Body Structures includes fewer items within the ICF-CY classification, the aspects it addresses, such as musculoskeletal and neuromotor abnormalities, are particularly impactful in our sample.

Furthermore, our analysis of mean frequency highlights that Body Functions, although relevant, appeared slightly less critical than Body Structures within this cohort. This suggests that anatomical abnormalities may have a greater direct impact on functional limitations than physiological dysfunction alone. Notably, social support emerged as one of the most influential factors. Existing literature already signalled social support as the most critical area influencing stress in these families, aligning with observations in families caring for chronically ill children [36–38]. Interestingly, some evidence found that the ability to cope with stress did not differed between families with SMA children and the others. Overall, the findings suggested that the emotional burden in families with SMA is primarily driven by disease severity, lack of social support, and to a lesser extent, child behaviour problems [39]. Some other aspects highlighted in the literature are strictly interconnected themes that capture the profound psychosocial impact of living with SMA on families [40]. The desire for their child’s independence, coupled with the constant uncertainty, helplessness, and significant financial strain brought on by caring for a child with SMA, creates a pervasive sense of stress for families [22].

Therefore, promoting social awareness focused on principles of inclusion is necessary to enhance the social connection potential for these patients [41]. The current study further underscores the critical role of social support networks. However, the data suggests that support from extended family, community members, people in positions of authority, and even pet therapy may not always be readily available to SMA patients [7]. The potential inadequacy of the existing social support system was evident in both the difficulties encountered in accessing public transportation or buildings and the lack of attention or commitment from authority figures. To enhance social recognition of this condition, two potential avenues for improvement could be: increasing training for healthcare professionals to cultivate greater social sensitivity towards this issue, and the development of a broader network that integrates the expertise of social workers.

The multidimensional approach, together with the implementation of the ICF-based assessment, enables the identification of the genuine needs of an individual, a human entity, transcending the restrictive notion of solely a “patient“ [32]. Gathering an individual’s true needs, encompassing those typically identified through the most relevant outcome measures and scales, constitutes the most authentic and practical approach to genuinely placing the patient at the core of the healthcare system. This principle is particularly significant in SMA, a condition characterized by distinct pathophysiological features. Traditionally, the identification of therapeutic objectives has prioritized structural interventions. However, our findings, consistent with the broader scientific literature, highlight the critical importance of addressing patients’ broader functional and psychosocial needs, extending beyond isolated impairments.

Despite its broad utility, implementing the ICF-CY framework requires specialized training, structured coding procedures, and interprofessional collaboration, which may pose logistical challenges in resource-limited settings. Additionally, integrating systematic ICF-CY assessments necessitates additional staff time, standardized workflows, and investments in training materials or digital infrastructure, factors that must be considered for broader implementation. Scaling this model across diverse healthcare settings may also be challenging due to variability in resources, institutional priorities, and staff expertise, emphasizing the need for adaptable strategies. Nevertheless, in the context of SMA’s evolving treatment landscape, the comprehensive insights provided by the ICF-CY remain crucial in ensuring that clinical advancements translate into meaningful improvements in patients’ daily lives.

### Limitations of the study

The primary limitation of this study is the small sample size (only 6 patients), which was determined by the number of consecutive admissions during the study period. This limitation is inherent to the design as a pilot study, which was intended to explore the feasibility of using the ICF framework for assessing SMA patients and to provide preliminary data that can inform larger, more robust studies. While the small sample size is understandable given the rare and neurodegenerative nature of SMA, it limits the generalizability of the findings. Results from this limited cohort may not fully represent the broader population of SMA patients, particularly those with different severity levels or from varied geographical or healthcare settings. Therefore, caution should be exercised when applying these results to the wider SMA population. However, to ensure data quality and mitigate sample-related constraints, systematic training of interviewers, a uniform protocol, and the use of comprehensive ICF-CY coding were employed throughout the study. Additionally, the use of the ICF as a classification tool presents another limitation. While it provides a broad qualitative framework for patient description, it lacks quantitative assessment capabilities. For a comprehensive evaluation, the ICF should be administered alongside validated clinical scales specific to SMA patients. Future research can address these limitations by expanding the sample size through collaboration with multiple institutions and by incorporating additional quantitative tools to complement the ICF.

## Conclusion

SMA requires a comprehensive, multidisciplinary approach to care. While SMN-targeted treatments are reshaping its natural course, significant gaps remain in disability management, necessitating more precise assessments of residual impairments. Beyond motor limitations, SMA affects multiple physiological domains, highlighting the need for holistic frameworks like the ICF, which evaluate both impairments and environmental influences. Measuring disability remains a critical challenge in refining care standards for SMA patients in the era of disease-modifying therapies. The ICF framework, integrated within a multidisciplinary approach, enables neurologists, physiatrists, pulmonologists, therapists, and psychologists to develop personalized interventions that address both functional and psychosocial needs. This patient-centered strategy fosters more effective care that enhances physical function, well-being, and quality of life.

## Data Availability

The authors confirm that the data supporting the findings of this study are available within the article.
